# Comparative population genetics of swimming crab host (*Portunus pelagicus)* and common symbiotic barnacle (*Octolasmis angulata*) in Vietnam

**DOI:** 10.7717/peerj.11671

**Published:** 2021-07-07

**Authors:** Binh Thuy Dang, Oanh Thi Truong, Sang Quang Tran, Henrik Glenner

**Affiliations:** 1 Institute for Biotechnology and Environment, Nha Trang University, Nha Trang, Khanh Hoa, Vietnam; 2 Department of Biological Science, University of Bergen, Bergen, Norway

**Keywords:** Symbiosis, Swimming crab, *Portunus pelagicus*, Barnacle, *Octolasmis*, Population genetics, Vietnam

## Abstract

**Background:**

By comparing spatial geographical structures of host populations with that of their symbionts light can be shed on their biological interactions, and the degree of congruence between host and symbiont phylogeographies should reflect their life histories and especially dispersal mechanisms.

**Methods:**

Here, we analyzed the genetic diversity and structure of a host, the blue swimming crab, *Portunus pelagicus*, and its symbiotic pedunculate barnacle *Octolasmis angulata* from six location sites representing three geographic regions (north, central and south) along the Vietnam coastline. High levels of congruence in their phylogeographic patterns were expected as they both undergo planktonic larval stages.

**Results:**

Based on the COI mtDNA markers, *O. angulata* populations showed higher genetic diversity in comparison with their host *P. pelagicus* (number of haplotype/individuals, haplotype and nucleotide diversity are 119/192, 0.991 ± 0.002 and 0.02; and 89/160, 0.913 ± 0.02 and 0.015, respectively). Pairwise Fst and AMOVA analyses showed a more pronounced population structure in the symbiotic barnacle than in its crab host. The DAPC analyses identified three genetic clusters. However, both haplotype networks and scatter plots supported connectivity of the host and the symbiotic barnacle throughout their distribution range, except for low subdivision of southern population. Isolation by distance were detected only for the symbiont *O. angulata* (R^2^ = 0.332, *P* = 0.05), while dbMEM supported spatial structure of both partners, but only at MEM-1 (Obs. 0.2686, *P* < 0.01 and Obs. 0.2096, *P* < 0.01, respectively).

## Introduction

The 3,260 km-long coastline in Vietnam is divided into the Gulf of Tonkin ecoregion in the North (South China Sea province), the central coast and the southeast coast (southern Vietnam ecoregion) and the Gulf of Thailand ecoregion in the South (Sunda Shell province) (Saito et al., 2004; Tran, 2006; Spalding et al., 2007). In these regions the surface current changes seasonally (North East-South West direction in the winter, while the ocean current flows in the South West-North East direction in the summer) (Pham et al., 2000; Pasuya et al., 2016).

The swimming crab, *Portunus pelagicus,* is common at all three locations where it is an economically important species for local fisheries. Throughout its entire distribution range, from the Indo-Pacific to the coast of Africa (Galil & Innocenti, 1999; Lai, Ng & Davie, 2010), the crab plays an ecological role as predator/prey/detritus feeder and as carrier of a variety of symbionts in shallow coastal and an island areas, especially mangrove forests, seagrass beds and coral reefs (Kunsook, Gajaseni & Paphavasit, 2014a; Kunsook, Gajaseni & Paphavasit, 2014b).

Symbiotic relationships, *mutualism, commensalism, or parasitism,* are common in marine ecosystems (Shields & Overstreet, 2003; Fountain et al., 2017; Nguyen et al., 2020) *and* reciprocal selection pressure between two (or multiple) interacting species can potentially alter the diversity, function and community dynamics of their hosts (Thrall et al., 2007; Thompson, 2010; Hadfield et al., 2014), as well as community structure, virulence, and transmission pattern of the symbionts *(Rigaud, Perrot-Minnot & Brown, 2010)*.

Ectosymbiotic species recorded on *P. pelagicus* (Shields, 1992; Alsaqabi, Eshky & Albelali, 2010; Shaharom & Ikhwanuddin, 2012), and the congeneric *P. sanguinolentus* (Le, Vo & Nguyen, 2018), can add up to ten different species, and the majority of crabs (65%) are infected by at least 3 ectosymbionts (Shields, 1992). One of the most common symbionts of *P. pelagicus* belongs to the pedunculate/stalked barnacle genus, *Octolasmis*, all species of which are minute, rarely more than 0.5 cm long, and attach to various body parts of the crabs (Jeffries et al., 2005; Kumaravel, Ravichandran & Rameshkumar, 2009; Machado et al., 2013). The symbiotic association between the about 30 accepted *Octolasmis* species (WoRMS, 2021) depends on the biological characteristics of the host such as distribution, sex, size, maturity stage, and molting cycle (Weng, 1987; Shields, 1992; Gaddes & Sumpton, 2004; Klinbunga et al., 2007; Babu et al., 2012; Machado et al., 2013). Although not having an individually negative effect on the host as e.g., parasitic barnacles or rhizocephalans (Isaeva, Dolganov & Shukalyuk, 2005; Mouritsen & Jensen, 2006; Amalia et al., 2016), a high individual density (mean intensity) of a species of *Octolasmis* is thought to hinder the host’s respiration and movement, and causing the host to change behavior (Jeffries et al., 2005; Machado et al., 2013; Waiho et al., 2017).

To establish symbiotic relationships, species have undergone a long-term coevolution processes, which should be considered within a community context (Thrall et al., 2007). The genetic population structure of host –symbiont has been reported to be affected by complex abiotic (seawater temperature, ocean currents, and geographic distance), and biotic (host population, symbiont transmission and virulent) factors (Huyse, Poulin & Théron, 2005; Dharmarajan et al., 2016). However, most of our knowledge is based on a few thoroughly researched host/parasite systems while limited research has focused on symbiotic non-model relationships such as crabs and parasites in consideration of their dispersal ability (Hay, Jorge & Poulin, 2018), and currently no studies have addressed the biological aspects of the association between crabs and symbiotic stalked barnacles.

This study aims to investigate the population genetic structure of the swimming crab *Portunus pelagicus* along the Vietnamese coastline and its common symbiotic barnacle *Octolasmis angulata* focusing on three questions: (1) Is there correlation between the phylogeography of the host and the symbiont? (2) Does isolation by distance affect the host-symbiont populations? (3) Is the spatial population structure of the host related to infestation rate of the symbiont?

## Material and Methods

### Host/symbiont sampling and identification

**Figure 1:**
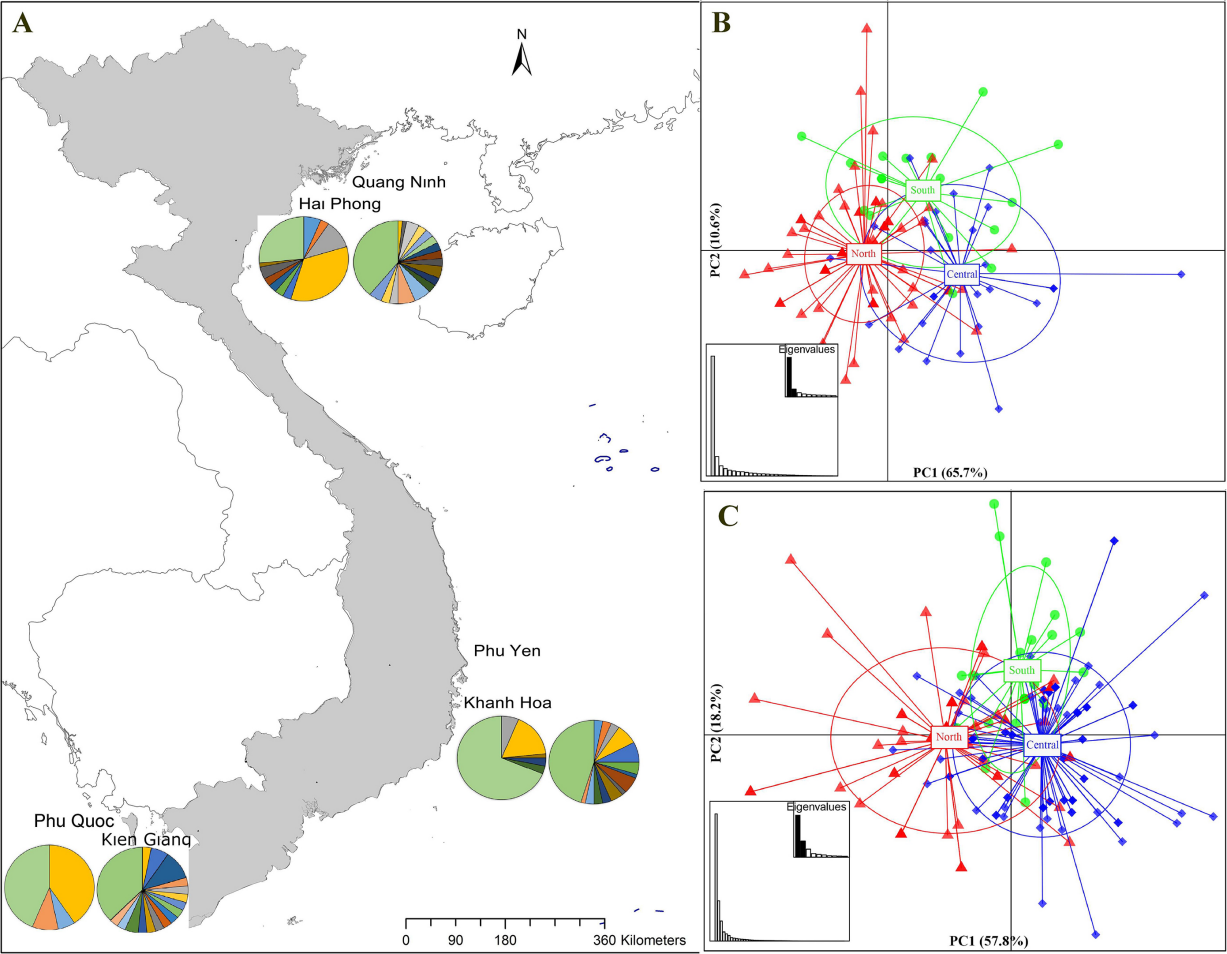
Sampling stations and population structure of *Portunus pelagicus* and *Octolasmis angulata* along the Vietnamese coastline using the COI mtDNA gene. (A) Sampling map and pie charts indicate the proportion of haplotypes at each site (*P. pelagicus* left and *O. angulata* right*)*. Colors represent the haplotypes shown in Fig. S1. (B) Scatter plot from DAPC of *P. pelagicus* and (C) *O. angulata.* Axes represent the first two Linear Discriminants (LD), the percentage of variability explained by each coordinate is shown in brackets. The circle represents clusters with 95% confidence limits and dots represent individuals.

The blue swimming crabs *Portunus pelagicus* (*n* = 160) were collected from fisherman at six locations representing three populations: north (Hai Phong-HP (*n* = 32) and Quang Ninh-QN (32), central (Khanh Hoa-KH (32) and Phu Yen-PY (32), and south (Kien Giang including Phu Quoc-PQ (16) and Rach Gia-RG (16)) of the Vietnamese coastline (Fig. 1A). The crabs were transferred to the laboratory, and freshly examined for ectosymbionts. The common pedunculate barnacles (*Octolasmis angulata*) were collected and identified following Jeffries et al. (2005) and Amalia et al. (2016). All of the crabs were coded following sampling sites, maturity and infestation status; and each barnacle from crab individuals were kept separately, and carefully marked. The *O. angulata‘s* individuals (*n* = 32) were chosen from each location (192 in total). Tissue samples (Chelipeds from crabs and whole body of barnacle) were taken from fresh specimens and preserved in 95% ethanol immediately after sampling.

### Molecular amplification

DNA was extracted with DNeasy Tissue Kit (Qiagen, Valencia, CA, USA) according to the manufacturer’s protocol. Part of the COI mtDNA region was amplified using primers LCO1490 and HCO2198 (Folmer et al., 1994). PCR reactions were performed in 50 µl with components as follows: 10 µL of Dream Taq buffer 10X, 2 µL dNTP (10 mM), 2 µL each primer (10 mM), 1.25 unit of Dream Taq polymerase (5U/µl), 5 µl DNA template and distilled water to the final volume. Amplification was implemented using the following PCR profile: a preliminary denaturation at 94 °C for 3 min, followed by 35 cycles of 94 °C for 45 s, annealing for 45 s 42 °C, and then 72 °C for 45 s. This was followed by a final extension period at 72 °C for 7 min before the samples were cooled to 4 °C. PCR products were run on 1.5% agarose gel for confirmation of equal length against an appropriate size markers. The PCR products were purified using DNA purification kits (Promega) and pre-sequenced using dye–labels dideoxy terminator (Big Dye Terminator 3.1, Applied Biosystems) with the same primer as the PCR reactions at the following temperatures: 96 °C for 30 s, 50 °C for 30 s and 60 °C for 4 min. Sequences of both strands were generated on an ABI PRISM 3100 Genetic Analyzer (Applied Biosystems) using the amplification primers. Sequences were saved in FASTA file format and multiple sequence alignment subsequently performed using CLUSTALX with default parameters (Thompson, Higgins & Gibson, 1994).

### Genetic diversity and Population structure

Genetic polymorphism was investigated by calculating haplotype diversity (gene diversity) and nucleotide diversity per site using DnaSP 5.10.01 (Rozas et al., 2003). Pairwise comparisons of Fst values between populations were computed in ARLEQUIN 3.11 (Excoffier, Laval & Schneider, 2017) with 100,000 iterations to test for significant differentiation among sampled sites. All *p*-values underwent FDR (False Discovery Rate) correction (Benajmini & Hochberg, 1995) to avoid false positives resulting from multiple comparisons. An analysis of molecular variance (AMOVA) was performed with 9,999 permutations and pairwise distant matrix using the program ARLEQUIN.

Haplotype networks were drawn with NETWORK 10.1.0.0 (Fluxus Technology, http://www.fluxus-engineering.com) by using the median joining algorithm (Bandelt, Forster & Röhl, 1999). Population structure was analyzed by Discriminant Analysis of Principal Components (DAPC) (Jombart, Sébastien & François, 2010) using the “adegenet” package (Jombart & Ahmed, 2011) in R. The *find clusters* function was used to detect the number of clusters in the population (the lowest associated Bayesian Information Criterion (BIC). This analysis provides a graphic description of the genetic divergence among populations in multivariate space.

To test whether the host and its symbiotic barnacle populations along the Vietnamese coastline are still connected by gene flow, we performed analyses of isolation by distance (both dispersal ability and migration rate) using mantel tests in the “vegan” package (Oksanen et al., 2016) implemented in R. The statistical significance of the parameter estimates was obtained via 9,999 permutations and 1,000 bootstraps resampling. According to Legendre & Fortin (2010) and Legendre, Fortin & Borcard (2015), the mantel test has proven to perform with low power in a spatial analyses, so the alternative distance-based Moran’s eigenvector maps (db-MEMs) analysis (Dray, Legendre & Peres-Neto, 2006) was performed using the R package “adespatial” (Dray et al., 2020) with 9,999 permutations. Inter-population geographic distances were directly calculated from latitude and longitude data using the ArcGis 10.1 (ESRI, 2011).

### Symbiont infestation and host distribution

The crab individuals (*n* = 160) represented geographical defined populations (North, Central and South) were categorized by their intensity: no infection (0), light (1–10 individuals/host), medium (11–50 individuals/host), and heavy (>50 individuals/host). The hierarchical infestation of *O. angulata* were plotted against the *P. pelagicus* populations to investigate the correlation between symbiont infestation pattern and host distribution.

## Results

### Genetic diversity and population structure

Across the three sampled populations, sequencing results for 160 individuals of *P. pelagicus* (GenBank accession number MN336861–MN337020, 658 bp) revealed 89 haplotypes. Haplotype number and diversity within sites ranged from 27 and 0.868 ± 0.037 (north) to 49 and 0.963 ± 0.015 (central), both sites for 64 individuals, 17 haplotypes/32 individuals and 0.835 ± 0.065 (south). Nucleotide diversity from 0.011 (south), 0.013 (north), to 0.019 (central). The southern population showed lowest genetic diversity, and the central population showed the highest genetic polymorphism in terms of haplotype and nucleotide diversity, and other parameters (Table 1).

**Table 1:** Summary statistics of genetic variation of *Portunus pelagicus* distributed in Vietnam coastline and symbiotic barnacle *Octolasmis angulata*.

**Population**	**N**	**Genetic diversity**					
		**N**_**h**_	**Hd**	*π*	**S**	*η*	**k**
***Portunus pelagicus***							
North	64	27	0.868 ± 0.037	0.013	35	35	8.897
Central	64	49	0.969 ± 0.015	0.019	54	58	13.168
South	32	17	0.835 ± 0.065	0.011	19	19	6.429
Total	160	89	0.913 ± 0.02	0.015	57	62	10.397
***Octolasmis angulata***							
North	64	42	0.981 ± 0.007	0.025	51	57	13.967
Central	64	43	0.985 ± 0.006	0.027	66	78	16.258
South	64	40	0.979 ± 0.008	0.023	57	64	14.973
Total	192	119	0.991 ± 0.002	0.027	92	114	16.211

**Notes.**

N number of individuals N_h_ number of haplotypes Hd haplotype diversity *π* nucleotide diversity S polymorphic sites *η* total number of mutations k average number of nucleotide differences

The barnacle populations were genetically more diverse than populations of the swimming crabs. Among 192 *O. angulata*, individuals, 119 haplotypes were defined (Accession number MN336669–MN336860, 672 bp). The haplotype diversity and nucleotide diversity of the pooled population of barnacles were 0.991 ± 0.002 and 0.002, respectively (Table 1). In congruence with the crab populations, the northern and central populations represented higher genetic diversity compared to the south (haplotype diversity and nucleotide diversity from 0.985 ± 0.006, 0.027; 0.981 ± 0.007, 0.02; and 0.979 ±0.008, 0.023, respectively) by (Table 1).

Pairwise Fst comparisons of the geographically defined crab populations varied from 0.015 to 0.058. Population differentiation was detected between the central and the southern population (*P* < 0.05), while no difference was found between the northern and the central, or the northern and the southern population (*P* > 0.05) (Table 2). Among the *O. angulata* populations, significant structuring was detected at all spatial levels (Fst from 0.058 to 0.128, *P* < 0.05), and Fst between the north and the central was smaller than those between north-south, and center-south populations (Table 2).

**Table 2:** Estimates of Fst between all populations of *P. pelagicus* (upper-half matrix) and *O. angulata* (lower-half matrix). Significance levels are indicated by the values at *P* < 0.05 are in bold.

	North	Central	South
North	–	0.035	0.015
Central	**0.058**	–	**0.057**
South	**0.128**	**0.117**	–

Hierarchical results of AMOVA (Table 3) revealed that 95.98% of the genetic variation occurred within populations and 4.02% of the variance contributed to differentiation across sample locations, suggesting high gene flow among crabs’ sampling locations. The symbiotic barnacle again showed a higher percentage of genetic differences between populations (10.19%), while 89.81% contributed to the within population variations, indicating that population structure is more pronounced in the symbiotic barnacle than its crab host.

**Table 3:** Analysis of molecular variance (AMOVA) of *Portunus pelagicus* and *Octolasmis angulata*.

**Source of variation**	**d.f.**	**Sum of squares**	**Variance components**	**Percentage of variation**	**Fixation index**	**P values**
***Portunus pelagicus***						
Among populations	2	31.85	0.21217	4.02	0.04	0.002
Within populations	157	794.719	5.0619	95.98		
Total	159	826.569	5.27407	100%		
***Octolasmis angulata***						
Among populations	2	124.505	0.855	10.19	0.102	<0.001
Within populations	189	1423.72	7.5329	89.81		
Total	191	1548.22	8.3879	100%		

The haplotype network of *P. pelagicus* revealed no population differentiation among the three populations (Fig. S1A). Only one haplotype (H4) appeared at all locations with high frequency, and 2 haplotypes (H3 and H27) were shared by at least two sites (Fig. S1A and Fig. S1B). The haplotype network of the symbiotic barnacle showed similar pattern of lacking population structure as the swimming crab (Fig. S1C). One haplotype appeared at most of the locations (H5). A few haplotypes (H6, H13 and H40) were shared by two sites. In both species, a high number of unique haplotypes (found at one location) were detected (75 out of the 89, and 78 out of 119 for *P. pelagicus* and *O. angulata*, respectively) (Figs. 1A and 1D).

The *find clusters* function indicated three clusters in both the host and the symbiotic barnacle populations (Figs. S2A & S2B). DAPC analyses using the detected number of clusters are shown in Figs. 1B and 1C. The eigenvalues (Fig. 1B, inset) showed that the genetic structure was captured by the first two principal components (65.7% and 10.4% of variances), which supported three groups of *P. pelagicus*. In a similar way, the first two PCs (57.8% and 18.3% of variance, respectively) of *O. angulata* DAPC plots (Fig. 1C) showed three overlapping clusters. In both cases, the southern population was slightly separated from the two remaining populations (central and north), and the trend is more clearly in the symbiotic barnacle.

**Figure 2:**
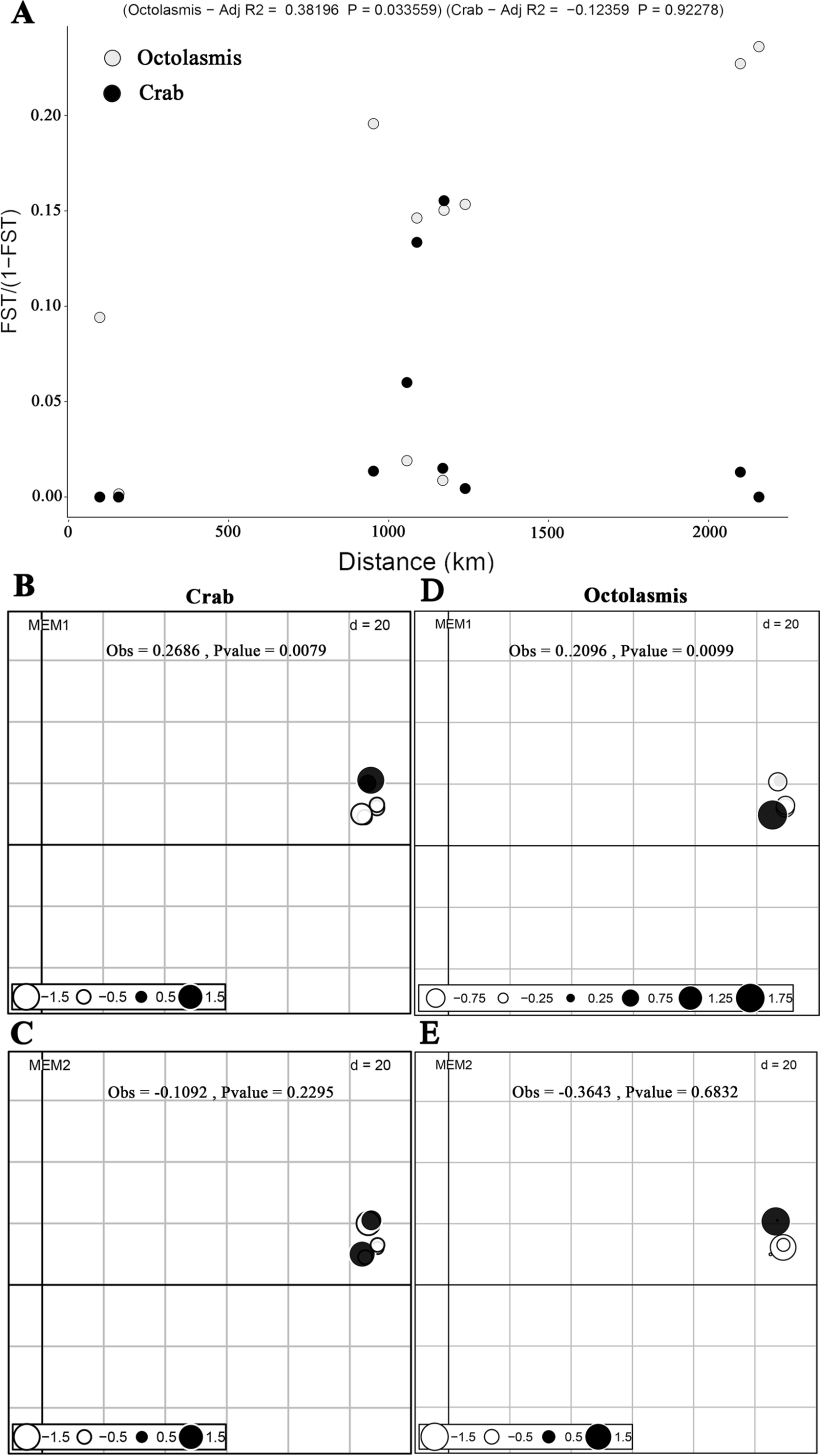
Scatterplots of Mantel test and dbMEM analysis. (A) Plotting pairwise Fst/1-Fst against pairwise geographic distances of crab host *Portunus pelagicus* and symbiotic barnacle *Octolasmis angulata* populations (100,000 permutations, *P* = 0.69 and *P* = 0.05 for host and symbiont, respectively); the first two dbMEM variables (Fst against pairwise geographic distances) of *P. pelagicus* (B & C) and *O. angulata* (D & E). The circles indicated the position and dbMEM values of each quadrat.

Based on COI sequencing data, we did not find isolation by distance (mantel test) for the *P. pelagicus* populations (R^2^ =−0.102, *P* = 0.69). This implies strong gene flow between crab populations along the Vietnamese coastline. The symbiotic barnacle, however, deviated from this pattern and isolation by distance was detected (R^2^ =0.332, *P* = 0.05) (Fig. 2A). dbMEM analysis, on the other hand, showed the spatial structure of both crab and barnacle (Figs. 2B and 2D), but only at MEM-1 (Obs. 0.2686, *P* < 0.01 and Obs. 0.2096, *P* < 0.01, respectively). From MEM 2–6, no positive spatial genetics were detected (*P* > 0.05) (Figs. 2C and 2E).

### Symbiont infestation and host distribution

**Figure 3:**
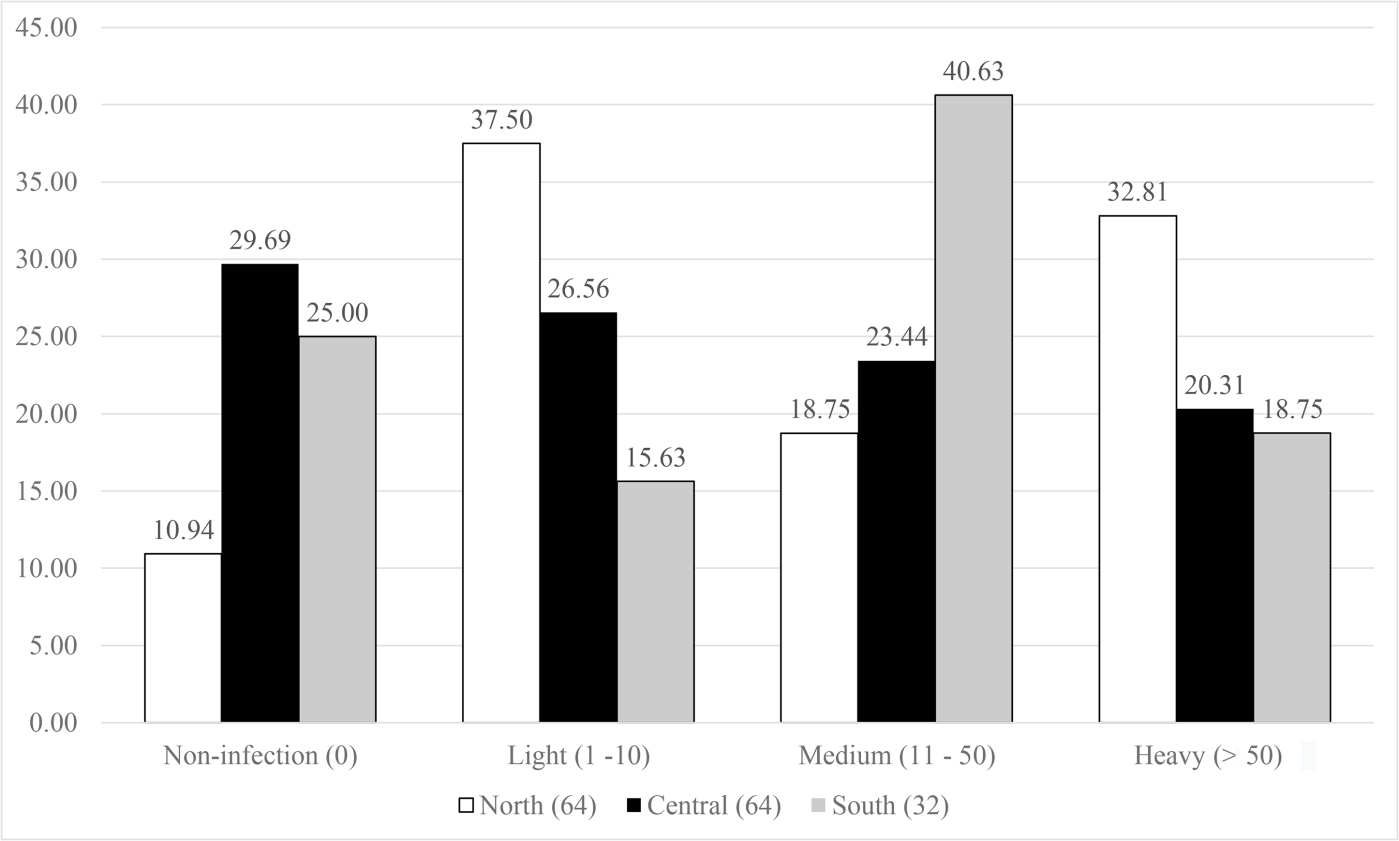
Infestation rates of the symbiotic barnacle, *Octolasmis angulata,* on the swimming crab host, *Portunus pelagicus* at three sampling locations. Results were based on 160 crab individuals following the hierarchical infestation rate as no infection (0), light (1–10 individuals/host), medium (11–50 individuals/host), and heavy (>50 individuals/host).

The symbiont infestation rates against host distribution are presented in Fig. 3. The northern population showed a great diversity in the infection levels, as the lowest proportion of the population (10%) had no infection, then for medium infection (18.75%), and high contribution to light (37.5%), and heavy (32.81%) infection. The central and south populations displayed a nearly equal percentage for both no- and heavy infected categories (29.69% and 25%, and 20.31% and 18.75% for no- and heavy infections, respectively). The south population showed highest percentage in medium infection (46.63%) and medium level for light infection (26.56%), while almost equivalent proportions (23.44–26.56%) were observed in the central population.

## Discussion

### Host and symbiont population structure

Our population genetic analyses demonstrated differences in population structure pattern between the crab, *P. pelagicus*, and its common symbiotic barnacle, *O. angulata*. In general, insignificant genetic structure was detected (Fst versus AMOVA, the haplotype network), suggesting high gene flow among the sampled crab populations along a more than 2,000 km long stretch of the Vietnamese coastline. The DAPC analysis did not indicate a spatial genetic pattern as no completely isolated cluster among three clusters was detected. In contrast, all populations of the barnacle symbiont, *O. angulata*, demonstrated significant structure differences (Fst = 0.056−0.128, *P* < 0.05; AMOVA 10.19%, Fst = 0.102, *P* < 0.01). However, neither haplotype network nor the DAPC analysis showed an obvious trend of population isolation. The southern population indicated a slight separation from the central and the northern populations, while more pronounced genetic mixing was observed between the central and the northern populations.

Using mitochondrial DNA and microsatellite markers, population connectivity was detected at the southeastern sea of China (Ren et al., 2018) and the Malaysia coastline (Chai et al., 2017) in congruence with *P. pelagicus* populations in Vietnam. A potential cryptic species complex of *P. pelagicus* (Lai, Ng & Davie, 2010) was indicated for the Chinese crab population, although heterozygote deficiencies due to inbreeding may explain the observation. However, using more sensitive SNP markers, Dang et al. (2019) discovered dis-connectivity and low gene flow in *P. pelagicus* along the Vietnam coastline, pointing to an isolated southern population. Different population structures by using different markers have been reported (Lemopoulos et al., 2019). However it is most likely that powerful and more polymorphic markers can better disclose subtle genetic variation than traditional markers (such as COI mtDNA) (Sorkheh et al., 2017) and substantiate the observed separation of the southern population of *P. pelagicus* in DAPC analysis of the present study.

The barnacles are a well-known crustacean taxon, whose diverse habitats range from organisms permanently attached to solid substrates as wood falls or rocky shores, organisms attached to other living organisms as epibionts (e.g., *Octolasmis*) to exclusively parasites (Rizocephala barnacle) and the taxon, Cirripedia, is often used as model for ecological and evolutionary research (Ewers-Saucedo et al., 2019). The intertidal acorn barnacle *(Chthamalus malayensis*) is a common and widely distributed species in Indo –West Pacific water (Southward & Newman, 2003). Phylogeographic analysis of *C. malayensis* have revealed low population subdivision, suggesting a high gene flow between the South China Sea (SC), Vietnam, and eastern Thailand. Here the barnacle populations are under the complex influence of the SC Warm Current, the mixing of Kuroshio Current, and the Southeastern Vietnamese Offshore (SVO) Current (Tsang et al., 2012). Unlike the free living intertidal barnacles, epibionts often undergo commensalism relationships https://en.wikipedia.org/wiki/Commensalism relationships by occupying the crab host’s shells (Fernandez-Leborans, 2010). However, in *Chenolibia testudinaria*, which, although less host specific, has approximately the same geographical distribution range as *O. angulata* (the West Pacific including Southern China, Taiwan, Southeast Asia, and Japan), no population differentiation was detected by Cheang et al. (2013). Rhizocephalan barnacles are exceptional parasites on various decapod crustacean hosts (Isaeva, Dolganov & Shukalyuk, 2005; Jung, Yoshida & Kim, 2019), and are well-known for their manipulation of the behavior and the morphology of their host (Høeg & Lützen, 1995). The rhizocephalan parasite on hermit crabs, *Peltogasterella gracilis*, is widely distributed throughout Korea, forms a single metapopulation with populations from Japan, although on different hermit crab species (Jung, Yoshida & Kim, 2019). The spatial genetic structure found in the present study in populations of *O. angulata* deviated from the high gene flow pattern of other South east-Asian barnacle species, and might be driven by diverse oceanographic factors. The Vietnamese coastal waters are known as a dynamic and complex system, characterized by monsoon-driven surface currents, different ocean systems (as the Gulf of Tonkin in the north, Gulf of Thailand in the south), the well-mixed East Sea, and the Pacific Ocean in the central regions (Pham et al., 2000; Chen et al., 2012; Pasuya et al., 2016). Dalongeville et al. (2018) suggested larval dispersal as the driving force to maintain gene flows at a small and intermediate spatial scale (<1,000 km), while geographic isolation explained genetic variation at a broader scale (>1,000 km) marine systems. Such processes may explain the genetic mixing of both *P. pelagicus* and *O. angulata* in the northern and central populations (approx. 1,000 km), and the slight splitting of the southern population (>1,500 km) in Vietnam. Additionally, upwelling and anticyclonic/cyclonic eddies along the south and central coasts (Chen et al., 2012) may cause larval retention, explaining the low genetic diversity, and weak subdivision in the crab and barnacle populations in southern Vietnam. The central populations showed high genetic diversity, and marked genetic mixing, which may be caused by the action of the Kuroshio and (SVO) currents, as in the case of *C. malayensis* (Tsang et al., 2012).

It would be logical if larval morphology and/ or larval development time could at least partially explain the differences in the geographical population patterns of the epibiontic barnacle species. An immediate expectation would be that larvae of *C. testudinaria*, which do not exhibit isolation by distance (IBD), have a greater dispersal potential than larvae of *O. angulata*, in which IBD apparently influences the population structure along the Vietnamese coastline. A greater dispersal potential could be achieved if larvae of *C. testudinaria* had a longer larval development time (which would allow them to follow the coastal water currents for longer periods, and over greater distances) and had morphologically more efficient swimming appendages. Rather counter intuitively, though, it turns out that *C. testudinaria* has an approx. three times faster larval development time (9 days) (Zardus & Hadfield, 2004) than *O. angulata,* which develops from nauplius 1 to the cypris larval stage in 31 days (Zardus & Hadfield, 2004; Yap et al., 2015)! The larvae of the two species, however, show large morphological differences. The nauplius larvae of *O. angulata* have, in contrast to *C. testudinaria* (a balanomorph barnacle), but like other lepadomorph barnacles (Jeffries et al., 1995), greatly enlarged frontal lateral horns, an extremely long dorsal thoracic spine and an extended thoraco-abdominal processes (Yap et al., 2015). It is conceivable that these structures change the hydrodynamics of the larvae and reduce the vertical sinking speed through the water column, which would lead to longer residence time in the current-carrying layers of the coastal water - and thus increase the dispersal capacity (Wong, Chan & Chan, 2018). It thus appears as if the larval development time and the larval morphology point in different directions, and it must be admitted that the morphological explanation for the difference in population structure between the two species is speculative. Future larval migration/transport studies will bring us closer to an understanding of the observed species-specific population differences.

For the crab host and its symbiotic barnacle, the different population patterns may reflect their life histories. Like other crustaceans, both *P. pelagicus* and *O. angulata* undergo long planktonic larval phases lasting for several weeks; 31 days in *O. angulata* (Yap et al., 2015), and between 20–40 days depending on the water temperature in *P. pelagicus* (Jose, 2015). To avoid fouling organisms, marine animals use a variety of ethological and physiological strategies such as molting, burying behavior, cleaning and motility (Fernandez-Leborans, 2010). Members of the barnacle genus *Octolasmis* exclusively settle on such semi-permanent substrata, which range from corals and arthropods to echinoderms and sea snakes (Jeffries & Voris, 1996). Because of the demand for integument of marine animals as obligate settling substrate, the development, from settled juvenile to a sexually mature adult barnacle, is known to be extremely fast in the genus. In the closely related *O. cor*, which uses the swimming crab *Scylla serrata* as host, adulthood of the barnacle is reached within 14 days (Jeffries, Voris & Yang, 1985), which entails that this species (and nothing indicates that this is radically different in *O. angulata*) can complete its lifecycle within the intermolt phase of even juvenile crabs with short molting intervals (Fernandez-Leborans, 2010). This is the case for *Octolasmis* species, which prefer the carapace of arthropods (not all *Octolasmis* species parasitize arthropods) as their settlement substrates where their survival relies on the ability to complete a life cycle within a single intermolt period of its host. Interestingly, Jeffries & Voris (1996) claim that “In several species (of *Octolasmis*) the cyprides are known to collect on crabs that are approaching ecdysis and to postpone settlement until the crab has completed moulting”. Successful dispersal of symbiont and parasitic species without or with restricted free-living stages often rely on the migratory capability of their hosts. Barnacles, however, possess extended pelagic larval stages (Kasten et al., 2019) and members of *Octolasmis* are no exception. This is an astonishing adaptation to a highly ephemeral settling substrate, crabs, which possess strong swimming ability, and, in order to spawn, or as a reaction to changing temperature or salinity, can move forth and back between estuaries and the open ocean (Kangas, 2000).

Due to the differences in life history and dispersal mode, it should be expected that the host exhibits a weaker population structure than the barnacle (limited by adult sessile phase). This prediction is supported by the current study. Additionally, isolation by distance (mantel and dbMEM analyses) seems to have contributed to local genetic differentiation between widely separated *O. angulata* populations. Hay, Jorge & Poulin (2018) did not find significant differences in population structure between isolated populations of a crab (*Hemigrapsus crenulatus*) and its acanthocephalan parasite (*Profilicollis novaezelandensis*), although the modes of dispersal in the two species are different (planktonic drift versus bird-mediated). The obscure biogeographic pattern of *O. angulata* due to mixed overlapping genetic clusters from the Haplotype network and the DAPC analysis, and supported local genetic differences (Fst and AMOVA analysis) calls for further studies.

### Symbiont infestation and host distribution

Symbiotic relationships affect the host in different ways (positively, neutrally, or negatively) and are the key drivers of ecological function and evolutionary processes (Fisher et al., 2017). A large body of research has documented that extended inter species relationships can often, but not always, lead to co-evolution (Song et al., 2015; Fountain et al., 2017; Pollock et al., 2018; Nguyen et al., 2020). The relationships between decapod hosts and species within *Octolasmis* appears to be a facultative rather than an obligatory association (Fernandez-Leborans, 2010), and there exists a large gap in our knowledge about population dynamics, and the driving forces leading to the relationships. However, the spatial distribution of host and symbiont is likely caused by tightly synchronized life cycles (Lion & Gandon, 2015), especially related to larval dispersal (Nishiguchi et al., 2008; Gibson, Jokela & Lively, 2016).

High infestation rate of fouling organisms such as barnacles have various negative effects (obstruct the respiratory and movement, change the behavior) on crustacean hosts (Gaddes & Sumpton, 2004; Machado et al., 2013; Amalia et al., 2016; Le, Dang & Tran, 2018). The fact that *O. angulata* is an extremely common symbiont, occurring on up to 90% of individuals in the host populations, and with intensities of more than 1,000 individuals (Shields, 1992; Khattab, 2017; Le, Dang & Tran, 2018), may indicate a long-term commensalism, and an effective strategy to successfully colonize and/or recolonize the host crabs. We plotted the infestation rates of *O. angulata* against the host distribution in order to investigate the correlation between symbiotic infestation and host spatial genetics. The high proportion of no infections observed in the central and south populations may reflect the limited dispersal of barnacle larvae due to anticyclonic/cyclonic eddies that cause larval retention (Chen et al., 2012; Teske et al., 2016). In contrast, complex monsoon driven currents in the Gulf of Tonkin (Pham et al., 2000) may support broad larval dispersal of both host and barnacle, subsequently resulting in low percentage of no and high levels of infestation. The southern population showed light and medium symbiotic infestation, while the northern population were light to heavily infected. According to Antonovics (2017), isolated host populations may facilitate symbiont virulence (meaning reducing host fitness) by favoring local transmission. If the southern crab population is isolated from the remaining populations, this may explain the higher symbiont infestation rate (light and medium infection) compared to the central and northern populations.

## Conclusions

Based on COI mtDNA markers, connectivity was observed in both the swimming crab, *P. pelagicus* and the symbiotic barnacle, *O. angulata* along the Vietnamese coastline. However, in contrast to the host, we found indications of differentiation in the symbiont populations between the three sampling areas, particular in the southern population.

##  Supplemental Information

10.7717/peerj.11671/supp-1 Supplemental Information 1 Haplotype network constructed of *Portunus pelagicus* (A) and *Octolasmis angulata* (C) using the COI mtDNA gene Each line represents one substitution; small unshaded circles indicate additional substitutions separating two haplotypes. The size of the circle is representative for the frequency of the haplotypes. Filled patterns correspond to geographic affinity of haplotypes. (B and D): Pie charts presents percentage of haplotypes contributed to the network following sampling sites for *P. pelagicus* (B) and *O. angulata* (D).

10.7717/peerj.11671/supp-2 Supplemental Information 2 Value of Bayesian information criterion (BIC) versus number of clusters for *Portunus pelagicus* (A), and *Octolasmis angulata* (B)

10.7717/peerj.11671/supp-3 Supplemental Information 3 Host and symbiont data The measurements of swimming crab (Portunus pelagicus) following the geographic locations, and the symbiotic status (number of symbionts), and the common symbiotic barnacle Octolasmis angulata (number of individuals).

## Additional Information and Declarations

### DNA Deposition

The following information was supplied regarding the deposition of DNA sequences: Data are available at Genbank: MN336861–MN337020 and MN336669–MN336860.

### Data Availability

The following information was supplied regarding data availability: The raw measurements are available in the Supplemental File.
