# Supplementary figures and images for: Comparative population genetics of swimming crab host (Portunus pelagicus) and common symbiotic barnacle (Octolasmis angulata) in Vietnam
Source: PeerJ. 2021 Jul 7;9:e11671. doi: 10.7717/peerj.11671 (PMC8272463; doi:10.7717/peerj.11671)

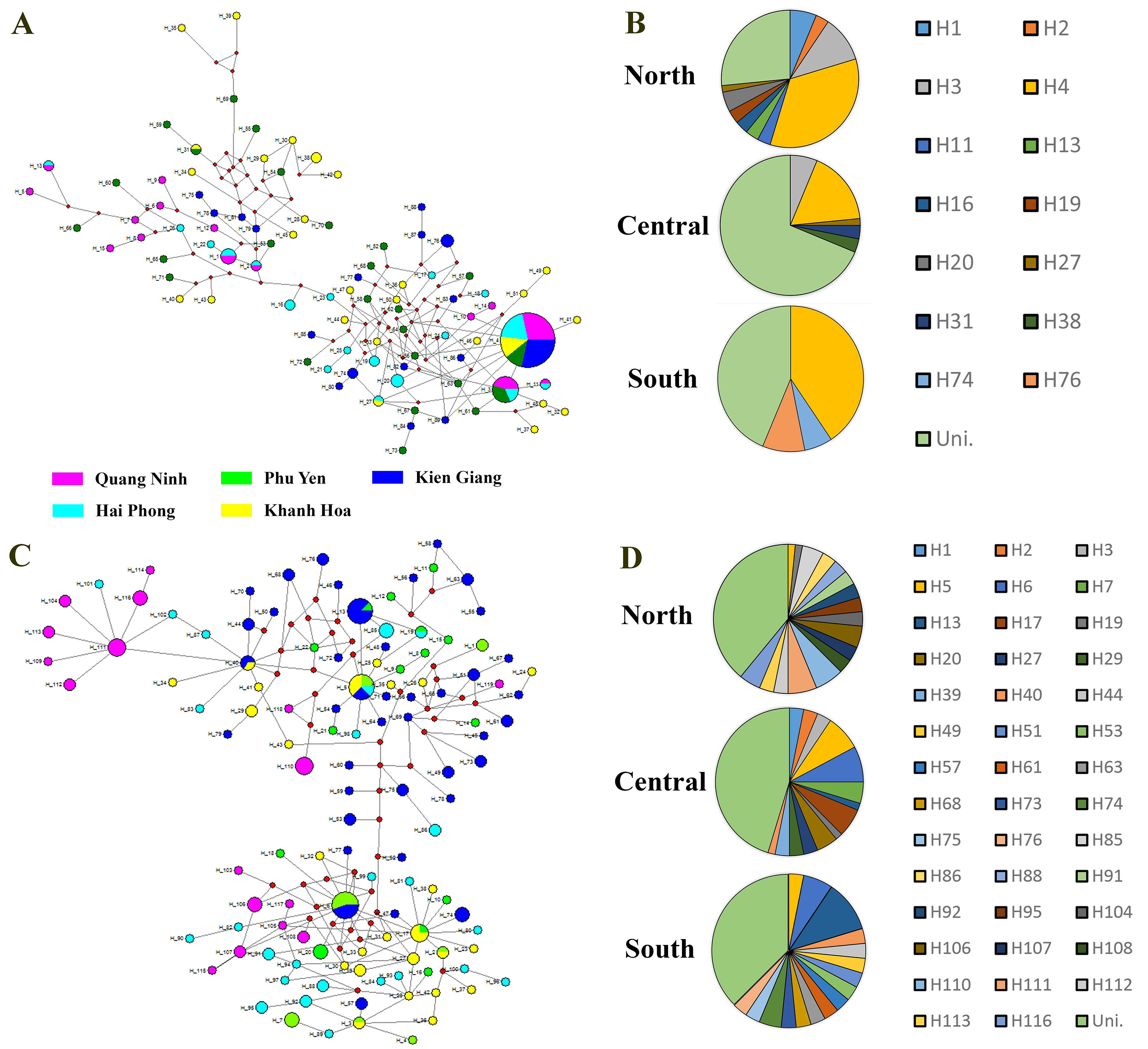

Supplement: Supplemental Information 1 — Each line represents one substitution; small unshaded circles indicate additional substitutions separating two haplotypes. The size of the circle is representative for the frequency of the haplotypes. Filled patterns correspond to geographic affinity of haplotypes. (B and D): Pie charts presents percentage of haplotypes contributed to the network following sampling sites for P. pelagicus (B) and O. angulata (D). [file peerj-09-11671-s001.png]

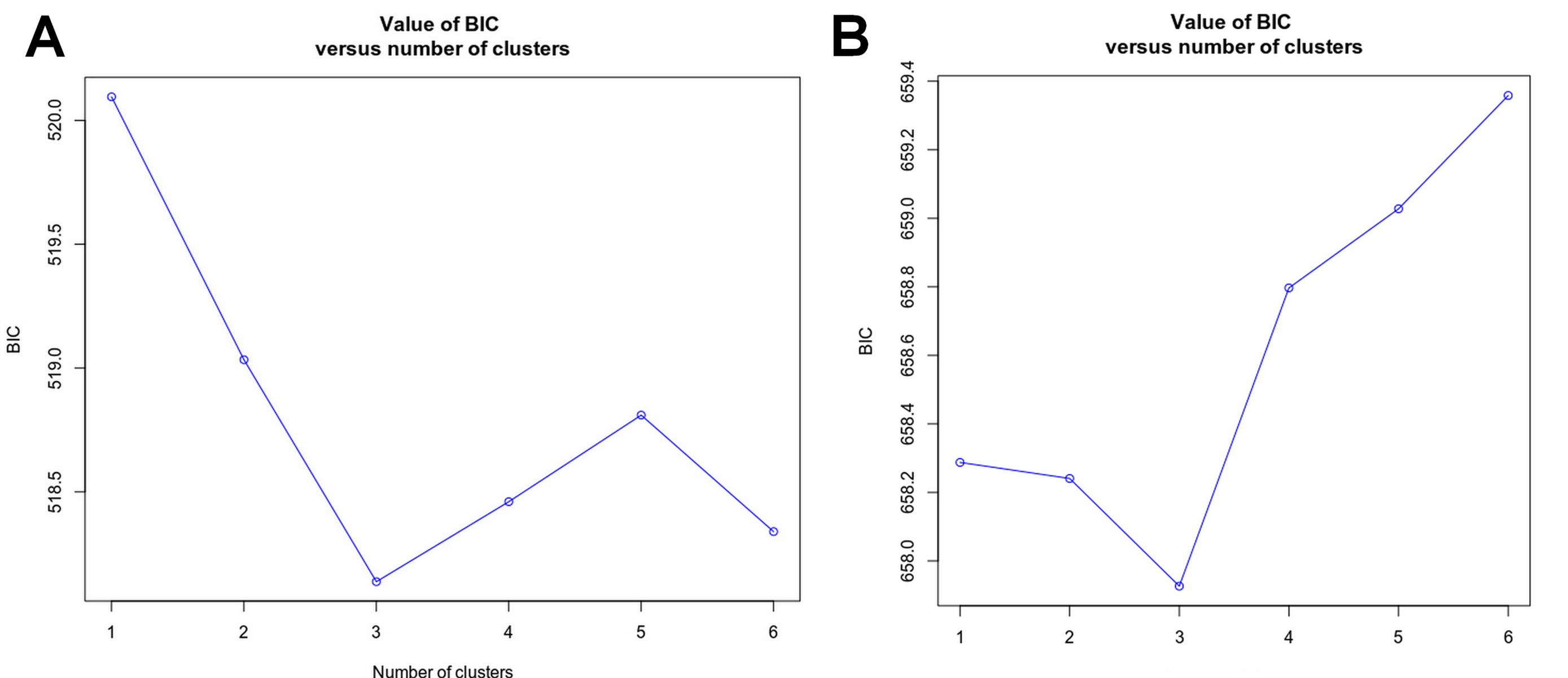

Supplement: Supplemental Information 2 [file peerj-09-11671-s002.png]
